# A process evaluation of the Supermarket Healthy Eating for Life (SHELf) randomized controlled trial

**DOI:** 10.1186/s12966-016-0352-3

**Published:** 2016-02-24

**Authors:** Dana Lee Olstad, Kylie Ball, Gavin Abbott, Sarah A. McNaughton, Ha N. D. Le, Cliona Ni Mhurchu, Christina Pollard, David A. Crawford

**Affiliations:** Centre for Physical Activity and Nutrition Research, Deakin University, 221 Burwood Highway, Burwood, VIC 3125 Australia; Deakin Health Economics, Deakin University, 221 Burwood Highway, Burwood, VIC 3125 Australia; National Institute for Health Innovation, School of Population Health, Tamaki Campus, University of Auckland, Auckland, 1072 New Zealand; School of Public Health, Faculty of Sciences, Curtin University, GPO Box U1987, Perth, WA 6845 Australia

**Keywords:** Process evaluation, Dietary behaviours, Fruits and vegetables, Carbonated beverages, Water, Food consumption, Food purchasing, Women, Supermarkets, RE-AIM

## Abstract

**Background:**

Supermarket Healthy Eating for Life (SHELf) was a randomized controlled trial that operationalized a socioecological approach to population-level dietary behaviour change in a real-world supermarket setting. SHELf tested the impact of individual (skill-building), environmental (20 % price reductions), and combined (skill-building + 20 % price reductions) interventions on women’s purchasing and consumption of fruits, vegetables, low-calorie carbonated beverages and water. This process evaluation investigated the reach, effectiveness, implementation, and maintenance of the SHELf interventions.

**Methods:**

RE-AIM provided a conceptual framework to examine the processes underlying the impact of the interventions using data from participant surveys and objective sales data collected at baseline, post-intervention (3 months) and 6-months post-intervention. Fisher’s exact, *χ*^2^ and t-tests assessed differences in quantitative survey responses among groups. Adjusted linear regression examined the impact of self-reported intervention dose on food purchasing and consumption outcomes. Thematic analysis identified key themes within qualitative survey responses.

**Results:**

Reach of the SHELf interventions to disadvantaged groups, and beyond study participants themselves, was moderate. Just over one-third of intervention participants indicated that the interventions were effective in changing the way they bought, cooked or consumed food (*p* < 0.001 compared to control), with no differences among intervention groups. Improvements in purchasing and consumption outcomes were greatest among those who received a higher intervention dose. Most notably, participants who said they accessed price reductions on fruits and vegetables purchased (519 g/week) and consumed (0.5 servings/day) more vegetables. The majority of participants said they accessed (82 %) and appreciated discounts on fruits and vegetables, while there was limited use (40 %) and appreciation of discounts on low-calorie carbonated beverages and water. Overall reported satisfaction with, use, and impact of the skill-building resources was moderate. Maintenance of newly acquired behaviours was limited, with less than half of participants making changes or using study-provided resources during the 6-month post-intervention period.

**Conclusions:**

SHELf’s reach and perceived effectiveness were moderate. The interventions were more effective among those reporting greater engagement with them (an implementation-related construct). Maintenance of newly acquired behaviours proved challenging.

**Trial registration:**

Current controlled trials ISRCTN39432901.

## Background

Unhealthy dietary patterns are among the leading public health concerns worldwide given their significant contribution to the global burden of disease [1]. Shifting dietary behaviours in health promoting directions is challenging however, given the ubiquity and relatively entrenched nature of unhealthy dietary patterns. In Australia, although most individuals ≥ 2 years of age consume fruits and vegetables (FV) on a daily basis, fewer consume sufficient quantities of fruits (54 %) and vegetables (7 %) for health benefits [2]. Intake of discretionary foods such as confectionary, cakes, sweet biscuits, pastries and soft drinks appear to be displacing intake of healthy foods such as FV within the Australian population, with more than one-third of daily energy intake contributed by these and other discretionary foods [2]. Strategies to promote purchase and consumption of FV and other healthy foods are therefore the subject of intense investigation.

Until recently, a paradigm of individual responsibility for health behaviours predominated within the nutrition community, such that many past investigations sought to improve dietary behaviours through increasing individual-level nutrition-related knowledge, motivation, and self-efficacy for healthy eating [3]. However, although intrapersonal factors are important correlates of food intake patterns [4–8], individual-level behaviour change interventions have generally yielded only modest outcomes [9, 10]. Indeed, behaviour change is not easily achieved nor maintained in the absence of structural supports, and therefore recent initiatives have sought to modify the context within which individual behaviours occur by creating environments that support healthy eating. Socioecological approaches integrate these diverse perspectives by acknowledging the joint contribution of individual, social and environmental factors to dietary behaviours [11, 12].

The current study is based on the Supermarket Healthy Eating for Life (SHELf) project, which was a randomized controlled trial (RCT) that operationalized a socioecological approach to population-level dietary behaviour change in a real-world supermarket setting [13, 14]. SHELf tested the impact of individual (skill-building), environmental (price reductions), and combined (skill-building + price reductions) interventions on women’s food purchasing and consumption behaviours. The study complemented past ecologically-informed supermarket-based RCTs performed in other nations. In a New Zealand study, 12.5 % price discounts on healthier foods led to increased purchasing of FV and other healthier foods, while in the Netherlands a 50 % discount on FV with and without additional education increased purchasing and consumption of FV [15, 16]. Education alone had no impact in either of these studies. Findings from the SHELf project differed somewhat from these earlier studies and were not always consistent with expectations. Specifically, in the immediate post-intervention period purchase of FV increased in the price reduction group, and purchase of fruit increased in the combined group, while no changes were found within the skill-building group. In addition, small unexpected increases in consumption of high-calorie carbonated beverages were observed in the skill-building and price reduction groups. Some of these behaviours were maintained at 6-months post-intervention, while others were not. These mixed and sometimes unexpected findings suggested a need to investigate the processes underlying these results.

Given the increasingly complex nature of public health interventions, and the need to evaluate their impacts in real-world contexts, it is no longer sufficient to confine evaluative activities to the realm of efficacy testing. A focus on program efficacy without attention to process contributes to a reductionist paradigm that oversimplifies reality and ignores essential details regarding why an intervention did or did not achieve its intended effects [17]. Process evaluation provides a means to monitor and document the implementation of health promotion programs in order to better understand why a program was or was not successful, and how any effects were achieved [18]. However despite its importance, few interventions within food shopping environments have undertaken process evaluation [19], and RCTs similar to SHELf have only examined selected aspects of reach and implementation [16, 20–23]. Limited availability of process-related data constrains efforts to apply lessons from past studies to improve the design and execution of future investigations. The purpose of this study was to conduct a process evaluation to investigate the reach, effectiveness, implementation, and maintenance of the SHELf interventions.

## Methods

### Participants and setting

Full methodological details of the SHELf study have been previously described [13, 14] and are briefly summarized here. SHELf was a socioecologically informed intervention conducted in two Coles® supermarkets (the second largest grocery chain in Australia) from May, 2011 to November, 2012. The Australian Bureau of Statistics’ Socioeconomic Index for Areas (SEIFA), derived from aggregate measures of social and economic information from the population census (such as the proportion of low-income households and the proportion of adults with a tertiary education) [24], was used to randomly select one advantaged and one disadvantaged neighbourhood that were serviced by a Coles supermarket within 25 km of Deakin University in Melbourne, Australia. Two target stores from within these areas were then purposively selected, and a random sample of 3000 women identified via customer loyalty cards (FlyBuys) who shopped regularly at these, or any other Coles stores with a 5 km radius, received a mail-out of a study recruitment pack. Women were targeted for recruitment because they tend to assume the majority of household food-related responsibilities [25, 26].

Women who responded to the mail-out (*n* = 693; 23 % response rate) were invited to participate if they met the following eligibility criteria: aged 18–60 years, the main household food shopper, shopped regularly at Coles supermarkets in one of the two catchment areas, held a Coles store loyalty card and were willing to use it when they shopped at Coles during the study period, able to speak, read and write in English, willing to disclose household income, and the only woman in their household taking part in the study (*n* = 674 women met inclusion criteria). The study was approved by the Deakin University Faculty of Health Human Ethics Advisory Group. All women provided written, informed consent prior to participating.

### Interventions

Following baseline measures (*n* = 32 did not return a baseline survey at all or in time to participate), 642 women were randomized using a computer-generated block randomization sequence into one of four study conditions: 1) Control (*n* = 161), 2) Price reductions (*n* = 161), 3) Skill-building (*n* = 160), or 4) Combined price reductions + skill-building (*n* = 160). The sample size provided 90 % power to detect an increase in vegetable consumption of at least 0.5 servings/day. Retrospective retrieval of 3 months of electronic purchasing data was followed immediately by a 3-month intervention period, with a subsequent 6-month no-intervention follow-up phase. Women in the control group did not receive any interventions during the study period, and were asked to maintain their usual shopping habits. The price reduction intervention aimed to reduce the cost of targeted healthier foods through 20 % price discounts on the purchase of all FV (including all varieties of fresh, canned, dried and frozen FV, excluding fruit juice, frozen hot chips and fried potatoes), low-calorie carbonated beverages, and water. Discounts were applied at the checkout counter upon swiping a FlyBuys card and were in addition to existing store promotions and discounts. Participants received a list of discounted items at the start and mid-way through the intervention. The skill-building treatment arm was developed on the basis of social cognitive theory and addressed theoretical constructs of nutrition-related self-efficacy, knowledge, and perceived facilitators and barriers for healthy eating through key messages provided in eight mailed newsletters and accompanying resources (e.g. recipes, goal-setting and self-monitoring exercises), and access to a dietitian-facilitated web-based forum that contained discussion boards and threads that coincided with newsletter content. Women in the combined price reduction + skill-building treatment arm received 20 % price discounts and all skill-building resources and supports.

### Theoretical framework

RE-AIM provides a conceptual model for understanding the impact of public health interventions [17, 27]. Overall impact is modelled as a function of five factors: reach, effectiveness, adoption, implementation, and maintenance [17]. We used RE-AIM as an organizing framework to investigate the reach, effectiveness, implementation and maintenance of the SHELf interventions (i.e. excluding adoption). This framework was selected because of its comprehensiveness, robustness demonstrated through several decades of use in a variety of fields, and adaptability to different purposes and contexts [27–29]. Kessler et al. [30] have outlined specific items across RE-AIM dimensions that represent comprehensive application of the full model. Our measures included these items with adaptations, additions and exclusions in some instances to accommodate the study design and availability of data. Factors at the organizational level (i.e. adoption, which refers to the proportion and representativeness of settings that adopt a program, along with maintenance of new programs at the organizational level) were not considered relevant given that study sites were pre-selected and were not expected to maintain the interventions beyond the study period.

### Data collection

Items for the process evaluation were assessed via completion of self-report surveys at baseline (T1), post-intervention (T2: 3 months) and 6-months post-intervention (T3: 9 months). We aimed to achieve a broad coverage of major issues relating to the impact of the interventions. It was not our intent to explore the hidden meanings underlying why participants did or did not find the interventions to be impactful, thus a brief survey was most appropriate to our aims. To promote retention, all participants received small gifts, including a A$20 shopping voucher for each of three survey completions, 1000 FlyBuys rewards points (approximately equivalent to A$15 value), and water bottles, shopping bags, spice packs and tea bags. Questions for the process evaluation were purpose-developed for the SHELf study and tailored according to treatment group assignment and contained a mix of closed- and open-ended questions. Core measures were identical on all surveys (e.g. sociodemographic details), however questions relating to specific elements of each intervention differed by intervention group (e.g. frequency of use of recipes, receipt of price discounts) as described below. The specific closed-ended questions asked and response options provided are presented in Tables 1, 2 and 3.

**Table 1:** Process evaluation of the skill-building intervention at post-intervention

Survey questions	Skill-building *n* = 160^a^ % (n)	Combined *n* = 160^a^ % (n)	P-value
Newsletters			
In the past 3 months, did you receive newsletters from us about healthy eating? (implementation)			0.493
* Yes*	98.0 (146)	99.3 (151)	
* No*	1 (2)	0 (0)	
* Don’t know*	0.7 (1)	0.7 (1)	
If yes, how useful did you find these newsletters? (implementation)			0.457
* 5 Extremely useful*	14.9 (21)	17.2 (25)	
* 4*	34.0 (48)	29.0 (42)	
* 3*	30.5 (43)	35.2 (51)	
* 2*	18.4 (26)	13.8 (20)	
* 1 Not at all useful*	2.1 (3)	4.8 (7)	
How many of the 8 newsletters did you read? (implementation)			0.919
* All*	51.8 (75)	51.7 (78)	
* About 75 %*	19.3 (28)	22.5 (34)	
* About 50 %*	16.6 (24)	13.3 (20)	
* About 25 %*	9.7 (14)	9.9 (15)	
* None*	2.8 (4)	2.7 (4)	
On average how long did it take you to read through a newsletter? (implementation) (mean ± SD)	11.9 ± 12.8 mins	11.8 ± 8.8 mins	0.899
Recipes			
Did you use any of the recipes provided? (implementation)			0.114
* Yes*	45.6 (67)	53.6 (81)	
* No*	53.1 (78)	46.3 (70)	
* Don’t know*	1.4 (2)	0 (0)	
If yes, how many have you used? (implementation)			0.860
* All*	1.5 (1)	2.4 (2)	
* 13–15*	1.5 (1)	1.2 (1)	
* 10–12*	0 (0)	1.2 (1)	
* 7–9*	1.5 (1)	4.9 (4)	
* 4–6*	30.8 (20)	24.4 (20)	
* 1–3*	64.6 (42)	64.6 (53)	
How often have you used the recipes? (implementation)			0.815
* Every day*	0	0	
* Every 2–3 days*	3.0 (2)	3.7 (3)	
* About once a week*	16.7 (11)	15.9 (13)	
* About once every 2 weeks*	34.9 (23)	28.1 (23)	
* About once a month*	19.7 (13)	28.1 (23)	
* Less than once a month*	25.8 (17)	23.2 (19)	
Did you find the additional materials useful? (e.g. the Food for Health brochures, Food Cents Eat Smart for 4 booklets, etc.) (implementation)			0.335
* Yes*	65.1 (97)	72.8 (107)	
* No*	18.1 (27)	12.9 (19)	
* Don’t know*	16.8 (25)	14.3 (21)	
Did you share any of the newsletters, additional materials or recipes with friends or family over the past 3 months? (reach)			0.853
* Yes*	30.4 (45)	33.1 (49)	
* No*	68.9 (102)	66.2 (98)	
* Don’t know*	0.7 (1)	0.7 (1)	
What do you think of the amount of materials (newsletters, recipes, and other materials) you received from us? (implementation)			0.969
* Too many materials*	21.0 (31)	21.9 (32)	
* About the right amount*	76.4 (113)	75.3 (110)	
* Did not receive enough*	2.7 (4)	2.7 (4)	

RE-AIM constructs examined are listed in brackets after each question

Fisher’s exact tests and *χ* ^2^ tests (for categorical data) and two-sided unpaired t-tests with unequal variances were conducted to assess differences in survey responses between treatment groups. Unadjusted multinomial logistic regression with robust standard errors was used to assess differences between groups where Fisher’s or *χ* ^2^ tests revealed overall differences

^a^Represents highest possible sample size for each question. The sample size for each question differs due to missing responses

**Table 2:** Process evaluation of the price reduction intervention at post-intervention

Survey questions	Price reduction *n* = 161^a^ % (n)	Combined *n* = 160^a^ % (n)	P-value
Did you receive discounts on fruit and vegetables as part of this study? (implementation)			0.157
* Yes*	84.8 (134)	78.4 (120)	
* No*	5.7 (9)	11.8 (18)	
* Don’t know*	9.5 (15)	9.8 (15)	
If yes, did you buy more fruit and vegetables because of the discount? (effectiveness)			0.474
* Yes*	65.4 (87)	62.7 (74)	
* No*	28.6 (38)	33.9 (40)	
* Don’t know*	6.0 (8)	3.4 (4)	
Did you receive discounts on diet/low-calorie soft drinks or water as part of this study? (implementation)			0.429
* Yes*	43.0 (68)	36.8 (56)	
* No*	30.4 (48)	36.8 (56)	
* Don’t know*	26.6 (42)	26.3 (40)	
If yes, did you buy more diet/low-calorie soft drinks or water because of the discount (effectiveness)			0.684
* Yes*	36.8 (25)	29.1 (16)	
* No*	58.8 (40)	67.3 (37)	
* Don’t know*	4.4 (3)	3.6 (2)	
If you received discounts do you think that you bought less sugar-sweetened/full calorie soft drinks because of the discount on diet/low calorie soft drinks? (effectiveness)			0.389
* Yes*	23.5 (16)	21.8 (12)	
* No*	61.8 (42)	70.9 (39)	
* Don’t know*	14.7 (10)	7.3 (4)	
Please indicate how much you liked or disliked the discounts you were offered (implementation)			0.457
* 5 I liked the discounts very much*	68.8 (108)	65.1 (99)	
* 4*	10.8 (17)	7.9 (12)	
* 3*	7.6 (12)	9.9 (15)	
* 2*	1.9 (3)	1.3 (2)	
* 1 I did not like the discounts*	3.2 (5)	2.0 (3)	
Did not receive	7.6 (12)	21 (13.8)	
If you received discounts, did you use the ‘supermarket items included in our 20 % price discount’ handout we gave you to help you work out your discount? (implementation)			0.193
* Yes*	53.1 (77)	45.8 (60)	
* No*	33.1 (48)	43.5 (57)	
* Don’t know*	13.8 (20)	10.7 (14)	
If you received discounts did you buy things for others using your discount? (reach)			0.450
* Yes*	13.8 (20)	11.5 (15)	
* No*	83.5 (121)	87.8 (115)	
* Don’t know*	2.8 (4)	0.8 (1)	

RE-AIM constructs examined are listed in brackets after each question

Fisher’s exact tests and *χ* ^2^ tests (for categorical data) and two-sided unpaired t-tests with unequal variances were conducted to assess differences in survey responses between treatment groups. Unadjusted multinomial logistic regression with robust standard errors was used to assess differences between groups where Fisher’s or *χ* ^2^ tests revealed overall differences

^a^Represents highest possible sample size for each question. The sample size for each question differs due to missing responses

**Table 3:** Process evaluation of the overall study at post-intervention

Survey questions	Control *n* = 161^a^ % (n)	Price reduction *n* = 161^a^ % (n)	Skill-building *n* = 160^a^ % (n)	Combined *n* = 160^a^ % (n)	P-value
Have you changed the way you buy, cook or eat food after taking part in this study? (effectiveness)					<0.001
* Yes*	14.7 (23)^b^	32.9 (52)^c^	34.5 (51)^c^	37.5 (57)^c^	
* No*	77.6 (121)	59.5 (94)	52.0 (77)	54.6 (83)	
* Don’t know*	7.7 (12)	7.6 (12)	13.5 (20)	7.9 (12)	
(If you have children aged 12 years of younger) Do you think your child/children are more willing to eat fruit and vegetables as a result of you taking part in this study? (reach)					0.227
* Yes*	15.4 (14)	26.0 (19)	22.1 (15)	29.7 (19)	
* No*	61.5 (56)	58.9 (43)	54.4 (37)	45.3 (29)	
* Don’t know*	23.1 (21)	15.1 (11)	23.5 (16)	25.0 (16)	
(If you have a partner) Do you think your partner is more willing to eat fruit and vegetables as a result of you taking part in this study? (reach)					0.017
* Yes*	18.1 (23)^d^	34.7 (42)^e^	30.6 (34)^e^	37.1 (43)^e^	
* No*	59.1 (75)	45.5 (55)	46.9 (52)	38.8 (45)	
* Don’t know*	22.8 (29)	19.8 (24)	22.5 (25)	24.1 (28)	

RE-AIM constructs examined are listed in brackets after each question

Fisher’s exact tests and *χ* ^2^ tests (for categorical data) and two-sided unpaired t-tests with unequal variances were conducted to assess differences in survey responses between treatment groups. Unadjusted multinomial logistic regression with robust standard errors was used to assess differences between groups where Fisher’s or *χ* ^2^ tests revealed overall differences

^a^Represents highest possible sample size for each question. The sample size for each question differs due to missing responses

^b,c^Values for variables without a common letter differ (*p* < 0.0001)

^d,e^Values for variables without a common letter differ (*p* < 0.05)

### Reach

Reach refers to the proportion of the target population that participates in an intervention and their representativeness [17]. Participants self-reported sociodemographic characteristics including age, highest educational qualification, household income, country of birth, marital status and number of children living in the home. Demographic characteristics of participants were compared to 2011 census data for the Greater Melbourne area [31] to determine the representativeness of participants. We additionally examined the reach of the interventions within participants’ social circles by asking whether they had shared skill-building intervention materials or price discounts with others, and whether participants thought their partner and/or children (where applicable) were more willing to eat FV as a result of their participation in the study.

### Effectiveness

Effectiveness refers to the positive and negative impacts of interventions on outcomes [17]. To supplement previously reported main outcomes from the SHELf study [13], this process evaluation assessed perceived positive and negative impacts of the interventions by asking those who accessed discounts whether the price reductions had caused them to purchase more of the discounted items, and if not, why this was the case, as well as how they had used any saved funds. All participants were asked whether they had changed the way they bought, cooked or ate food as a result of study participation, and to qualitatively describe any changes they had made.

### Implementation

Implementation is a multi-dimensional construct that refers to what a program consists of when it is delivered [32].

#### Skill-building

Participants were asked whether they had received newsletters, how many they had read, how useful they found them, and the average time taken to read one. Participants were also asked to qualitatively describe reasons why they may not have read all of the newsletters, the main messages they recalled from reading them, and the most and least useful parts. With respect to recipes, participants reported whether they had used recipes, how many they had used, and how often. Those who had not used recipes were asked to describe why this was the case. Finally, participants indicated whether they found the additional skill-building materials (i.e. brochures, booklets) useful, and whether the volume of skill-building materials provided was appropriate.

#### Price reductions

Participants reported whether they had accessed price discounts on FV and on low-calorie soft drinks and water. A subsequent question asked whether participants had ever consulted the list of discounted items they had been given. Participants were also requested to quantitatively rate and qualitatively describe the degree to which they liked the price reductions overall.

#### Overall

Participants were asked to describe what they liked most and least about the study.

#### Impact of intervention dose

Two dose-related variables were selected for each of the skill-building and price reduction interventions to evaluate whether the dose of the interventions received influenced purchase and consumption of FV and beverages at T2 (immediately post-intervention). Within the skill-building intervention, dose-related variables selected were those where there was sufficient variability in responses (e.g. we did not consider whether participants had received newsletters as nearly 99 % reported receiving them), and where responses were characterized by meaningful cut-points (e.g. number of recipes used was not examined as most participants used 1–3 or 4–6 recipes and the difference between these was not theoretically meaningful). Thus, the dose-related variables analysed were: number of newsletters read (100 % vs < 100 %), use of recipes (yes vs no), and whether price discounts on FV (yes vs no) and beverages (yes vs no) were accessed. Outcomes for the analysis included purchase (grams/week) and consumption (servings/day) of FV (including all varieties of fresh, canned, dried and frozen FV, excluding fruit juice, frozen hot chips and fried potatoes), and purchase (millilitres/week) and consumption (servings/day) of carbonated beverages and water. As described in a previous publication, consumption data were self-reported via a Food Frequency Questionnaire that used validated questions, while purchasing outcomes were assessed using electronic sales data obtained from customer store loyalty cards [13].

### Maintenance

Maintenance measures the extent to which newly acquired behaviours are maintained over time [17], and was assessed at T3 (6-months post-intervention).

#### Skill-building

Participants were asked whether they had continued to use any study materials in the post-intervention period, to report the number of materials used, and the frequency with which they had used them. Participants also indicated whether they had shared any intervention materials with friends/family in the past 6 months.

#### Price reductions

Participants were asked whether they had changed their purchasing of FV and of soft drinks and water in the past 6 months.

#### Overall

Participants reported whether they had changed the way they bought, cooked or ate food as a result of the study, and whether their partner and/or children (where applicable) were more willing to eat FV as a result of their involvement in the study.

## Data analyses

### Statistical analyses

Data from all participants who completed any portion of the T2 (*n* = 619) and T3 (*n* = 608) surveys were included, thus the sample size differed for each question. Fisher’s exact tests and *χ*^2^ tests (for categorical data) and unpaired t-tests with unequal variances (for continuous data) were conducted to assess differences in survey responses between treatment groups. Unadjusted multinomial logistic regression with robust standard errors was used to assess differences between groups where Fisher’s or *χ*^2^ tests revealed overall differences, with ‘no’ as the reference category.

Linear regression analyses assessed differences in FV and beverage purchasing and consumption at T2 according to the dose-related variables selected for the analysis. Individuals in the combined group were pooled with those in the price reduction or skill-building groups for interventions that both groups received (e.g. those who accessed price reductions on FV in the price reduction and combined groups were pooled, those who used recipes in the skill-building and combined groups were pooled, etc.) to assess the impact of all four dose-related variables (i.e. number of newsletters read, use of recipes, whether price discounts on FV and beverages were accessed). All models controlled for baseline values of the outcome and intervention group, and for the following a priori-determined covariates: age, country of birth, catchment area, marital status, household income, and the number of children living at home. Given the skewed distributions of several outcomes, bootstrapping with 1000 resamples was used to produce more robust standard errors. Participants who withdrew from the study (*n* = 3) and who were missing main outcomes from T1 (*n* = 10) or T2 (*n* = 30) were excluded. Participants who responded ‘don’t know’ (see numbers in Tables 1 and 2) to individual questions were additionally excluded from these analyses, as the dose received was uncertain in these cases.

All statistical tests were two-sided and the significance level was set at *p* < 0.05. Stata software (version 13; StataCorp LP, TX) was used for all statistical analyses.

### Qualitative data analysis

Qualitative data were coded in an inductive manner by a single investigator using thematic analysis procedures [33]. NVivo software (version 10.0; QSR International, Doncaster, VIC, Australia) was used to organize the data.

## Results

We present quantitative findings first, followed by qualitative findings which provide context for, and help to explain the reasons underlying the quantitative results. We chose to summarize qualitative responses rather than to present specific quotations given the brief nature of the open-ended responses provided. Full quotations would not have provided additional information in this instance.

### Reach

#### Quantitative findings

A total of 642 of the 3,000 women identified by Coles supermarkets as shopping at least once every 2 weeks within the target stores or any other Coles within a 5 km radius were randomized. Three individuals actively withdrew from the study following randomization, thus 21.3 % of the target population participated. Notably, this proportion might have been higher had we devoted additional efforts to enrolling additional non-responders. Demographic characteristics of participants have been previously presented [13]. Participants were nearly evenly split according to neighbourhood-level socioeconomic status (SES), with 44 % of participants residing in low, and 55 % in high SES areas [13]. Comparison of participant characteristics with the general population of females living in the Greater Melbourne region showed that a higher proportion of women enrolled in SHELf were born in Australia (71 % vs 63 %), and compared to the population of females age ≥ 20 years, a greater proportion of women in SHELf were married (71 % vs 55 %), and had less than a high school education (12 % vs 35 %). Compared to all households in Melbourne, a lower proportion of women in SHELf had a household income < $52,000 AUD/year (24 % vs 34 %). Self-reported consumption of fruit (1.9 servings/day) and vegetables (2.5 servings/day) was higher than the Australian average of 1.0 servings/day and 2.2 servings/day, respectively, among women ≥ 19 years [2].

The study’s reach extended to some of the families and friends of participants. Just under one-third of participants who received skill-building materials shared them with family/friends (Table 1), while 13 % of those who reported they had accessed the price discounts shared them with others (Table 2). Compared to individuals in the control group, participants in the intervention groups reported that their partners were more willing to consume FV as a result of their involvement in the study (*p* < 0.05), although their children were not (Table 3).

## Effectiveness

### Price reductions

#### Quantitative findings

Nearly two-thirds of participants who reported that they had accessed price discounts on FV said they purchased more FV because of the discounts, while the reverse was true among those receiving discounts on low-calorie carbonated beverages and water, with approximately two-thirds reporting they did not purchase more low-calorie carbonated beverages and water, or fewer high-calorie carbonated beverages because of the discounts (Table 2).

#### Qualitative findings

Those who did not increase their purchasing of FV said they had not done so because they already purchased what they required and additional purchases would have gone to waste. Others said they already purchased their FV elsewhere, often for reasons related to perceived cost and/or quality. Notably, a number indicated that although they had not increased their overall FV purchasing, they were now buying a greater proportion of their FV at Coles to take advantage of the price discounts they received through the study. Themes that emerged with respect to why so many participants did not increase purchasing of low-calorie carbonated beverages and water in response to price discounts related to the fact that many participants tended not to buy these beverages, often because they preferred tap water or wanted to avoid artificial sweeteners. Other participants indicated that they did not increase purchasing of discounted beverages simply because they did not require more of them.

Participants were asked to report how they used the funds they saved from the FV and beverage discounts. Many reported using them to purchase more food overall, with a large constituency indicating they spent these funds on FV specifically. Very few used the money to purchase additional discounted beverages. Others used their savings to fund other household-related expenditures, while still others used the money to purchase ‘luxury’ items they would not normally buy, such as more expensive types of FV, restaurant meals or magazines. One participant used the funds to purchase cigarettes. This was the only instance in which it was possible to ascertain that saved funds had clearly been used to purchase unhealthy items. Finally, some participants did not spend the saved funds or were unsure how they had used them, often indicating this was because the savings were too small to notice.

### Overall

#### Quantitative findings

Compared to those in the control group (15 %), significantly more participants in the intervention groups (35 %) said they had changed the way they bought, cooked or ate food as a result of study involvement (*p* < 0.001), with no differences among intervention groups (Table 3).

#### Qualitative findings

Reported changes made as a result of study involvement were very consistent, as participants overwhelmingly indicated that they had increased their purchase and/or consumption of FV, and of vegetables in particular. Many said they had increased the variety of foods they ate, especially of FV, and more often purchased these items fresh. Very few participants reported changing their beverage purchasing or consumption patterns as a result of study involvement. Some participants described changes they had made in more general terms, saying they had increased healthy, and reduced unhealthy eating. Additional changes made primarily by those who received skill-building materials included planning meals and shopping trips more often, incorporating FV within recipes, and increased awareness of food choices, although these types of changes were also reported by individuals in the price reduction and control groups. Individuals who received price discounts also said they had increased purchasing of FV at Coles to take advantage of the price discounts. Only three participants suggested the possibility of unintended negative consequences, including eating more unhealthy food or feeling guilty about eating unhealthy foods during the intervention period, although it was not clear whether they attributed these to the interventions specifically.

## Implementation

### Skill-building

#### Quantitative findings

Nearly all participants randomized to receive skill-building materials reported receiving newsletters, with more than half reading all of them (Table 1). On average, participants spent just under 12 min reading each newsletter. On a 5-point scale where 5 indicates ‘extremely useful’ and 1 indicates ‘not at all useful’, 80 % of participants who reported receiving newsletters gave them a score of 3 or higher. Although participants said they appreciated the recipes within the newsletters, only approximately half reported using the recipes provided, with most using only 1–3 recipes and doing so on an infrequent basis (i.e. no more than once every 2 weeks). Overall, three-quarters of participants thought the amount of material provided in the skill-building intervention was ‘about right’.

The majority of participants (85 %) signed up to participate in the web-based forums. Of those participants who signed up, 25 % logged onto the forums at least once, although only 10 % actively posted on the forums during the intervention period.

#### Qualitative findings

The vast majority of those who did not read all eight newsletters failed to do so due to time constraints, with many indicating they had put them aside to read later. Others indicated that they had not read all of the newsletters because the information they contained was too simplistic and they already knew much of it. Participants overwhelmingly appreciated the recipes that newsletters provided. Many also liked the information about FV and sections providing practical tips and time-efficient strategies for healthy living. Most participants said there was nothing they disliked about the newsletters. The primary messages they recalled from reading newsletters were often very general messages emphasizing the importance of eating healthfully, eating more FV, and healthy lifestyles.

Most of those who did not use the recipes had not done so because they already had a collection of recipes they enjoyed and were accustomed to using, which in some cases were similar to those provided by SHELf. Others indicated they had not had the time to use the recipes, while many said they did not find the recipes appealing, primarily because they were too basic. A number said the recipes did not fit with their current diet (followed for cultural, medical or lifestyle-related reasons).

### Price reductions

#### Quantitative findings

More than 80 % of individuals in the price reduction groups reported accessing discounts on FV, with just under 40 % reporting they had accessed the beverage discounts (Table 2). More than two-thirds liked the collective discounts ‘very much’.

#### Qualitative findings

Aspects of the discounts that participants most enjoyed were that they allowed them to save money, primarily on FV, with very few mentioning the beverage savings. Many indicated the discounts allowed them to buy more and/or a greater variety of FV, particularly the more expensive types. Participants said that receiving discounts made them feel appreciated and like they were being rewarded for making healthy choices. A number also indicated that they shifted their FV purchasing from other stores to Coles to take advantage of the discounts.

Participants expressed very few complaints related to the discounts, and were disappointed when they ended. Many wished the discounts had applied to more, or even all supermarket items. Several indicated they did not like the beverage discounts because they were not useful for them. Some indicated the size of the discounts was too small, and that even with the discounts they could still purchase less expensive produce elsewhere.

### Overall

#### Qualitative findings

When participants were asked what they liked most about the study, increased awareness of current eating behaviours was a strong theme across all groups, including those in the control group. This was attributed to the chance for self-reflection through completing surveys, information provided in the skill-building intervention, as well as through the foods to which discounts were applied. Appreciation for the incentives and discounts provided through the study emerged as another strong and consistent theme. Many participants also valued the opportunity to contribute to research, as they found the study interesting and worthwhile, and enjoyed completing the surveys. Those in the skill-building arms found the newsletters and recipes helpful and liked learning new things. The vast majority of participants indicated that they did not dislike anything about the study. The small number of participant-expressed concerns related primarily to the surveys, as some disliked the repetitive nature of the questions, the length of the surveys, or that they could not always find their preferred response options. Others disliked the non-monetary incentives, and felt this money could have been better spent elsewhere. Complaints related to the discounts were rare, whereas concerns were expressed with the newsletters, in terms of their messaging, the frequency with which they were received (i.e. too frequent), and their length (i.e. too long). Finally, environmental concerns were raised regarding the amount of packaging required to post non-monetary incentives, and provision of paper-based newsletters and surveys.

### Impact of intervention dose

#### Quantitative findings

In adjusted analyses, individuals who said they read all of the newsletters had a higher intake of low-calorie carbonated beverages and water (0.97 servings/day, 95 % CI 0.34–1.60; *p* < 0.01) compared to those who said they did not read all of the newsletters immediately post-intervention, while those who reported using the provided recipes had a higher intake of fruits (0.24 servings/day, 95 % CI 0.03–0.45; *p* < 0.05) and vegetables (0.27 servings/day, 95 % CI 0.05–0.49); *p* < 0.05) compared to those who did not use these recipes (Table 4). Those who reported accessing the FV discounts purchased (518.86 g/week, 95 % CI 158.41–879.31; *p* < 0.01) and consumed (0.48 servings/day, 95 % CI 0.13–0.84; *p* < 0.01) more vegetables compared to those who said they did not access discounts on FV. Those who said they accessed beverage discounts purchased more low-calorie carbonated beverages and water (1004.24 mL/week, 95 % CI 134.44–1874.03; *p* < 0.05) compared to those who said they did not access these discounts.

**Table 4:** Impact of intervention dose on purchase and consumption of fruits, vegetables and beverages at post-intervention

Dose-related variables	Fruit purchase (g/week)	Fruit consumption (servings/day)^a^	Vegetable purchase (g/week)	Vegetable consumption (servings/day)^a^	Low-calorie carbonated beverage and water purchase (mL/week)	Low-calorie carbonated beverage and water consumption (servings/day)^a^	High-calorie carbonated beverage purchase (mL/week)	High-calorie carbonated beverage consumption (servings/day)^a^
	**β (95 % confidence interval)**							
**Number of newsletters read** All vs < all^b^ (*n* = 285)	2.49 (−241.77, 246.75)	−0.06 (−0.27, 0.14)	113.82 (−103.66, 331.31)	0.06 (−0.17, 0.30)	188.98 (−502.78, 880.74)	0.97** (0.34, 1.60)	507.94 (−407.14, 1423.02)	−0.07 (−0.16, 0.01)
**Used recipes** Yes vs no^b^ (*n* = 282)	128.72 (−114.44, 371.88)	0.24* (0.03, 0.45)	−13.87 (−233.24, 205.50)	0.27* (0.05, 0.49)	248.27 (−317.73, 814.26)	0.38 (−0.24, 1.00)	95.23 (−635.92, 826.38)	−0.05 (−0.16, 0.06)
**Accessed price discounts on fruit and vegetables** Yes vs no^b^ (*n* = 274)	238.93 (−152.89, 630.75)	0.28 (−0.08, 0.63)	518.86** (158.41, 879.31)	0.48** (0.13, 0.84)	n/a	n/a	n/a	n/a
**Accessed price discounts on beverages** Yes vs no^b^ (*n* = 221)	n/a	n/a	n/a	n/a	1004.24* (134.44, 1874.03)	0.48 (−0.23, 1.19)	1074.80 (−656.43, 2806.03)	−0.02 (−0.13, 0.08)

Beta coefficients represent the between-group mean differences in purchasing and consumption of fruits, vegetables and beverages. Linear regression models controlled for baseline values of the outcome, intervention group, age, country of birth, catchment area, marital status, household income, and the number of children living at home

^a^1 serving of fruit = 150 g; 1 serving of vegetables = 75 g; 1 serving of beverage = 125 mL

^b^Indicates reference category

*p < 0.05; **p < 0.01; bolded values highlight significant differences

## Maintenance

### Skill-building

#### Quantitative findings

Nearly 44 % of individuals who received skill-building materials reported using them in the 6-month post-intervention period, with 32 % using newsletters and 47 % using recipes, albeit on an infrequent basis (i.e. no more than once every 2 weeks). Only 23 % reported sharing skill-building materials with family/friends in the post-intervention period.

### Price reductions

#### Quantitative findings

A minority (27 %) of those who said they had accessed price reductions during the intervention period reported that they changed their purchasing of FV after the price reductions stopped, while even fewer (9 %) reported that they had changed their beverage purchasing patterns in this period.

### Overall

#### Quantitative findings

At the 6-month follow-up, similar proportions of individuals in all groups (31 %) reported that the study had influenced the way they purchased, cooked or ate food. There were no differences among groups in the proportion of participants who reported their partners (31 %) and/or children (28 %) were more willing to eat FV as a result of their involvement in the study.

#### Qualitative findings

With respect to their maintenance of intervention-promoted behaviours, participants described attempts to increase healthy eating, primarily through including more or a greater variety of FV in their diets, and to drink more water. Participants also mentioned attempts to reduce unhealthy eating, such as through drinking fewer sugar-sweetened beverages. Many had become more aware of the importance of healthy eating. Others described more frequently planning their meals and shopping, and using more, or a greater variety of recipes.

## Discussion

SHELf was a RCT that compared the impact of individual, environmental and a combined approach to promoting healthy eating in the supermarket setting, a key environment for influencing food selection and consumption [34]. Process evaluation was undertaken with the aim of elucidating factors implicated in the study’s previously reported main outcomes [13]. The RE-AIM framework proved valuable in guiding this analysis, and in collating the most consequential and policy-relevant findings. Overall, the reach of the SHELf interventions to disadvantaged groups, and beyond study participants themselves was moderate. Participants indicated limited appreciation for, and use of beverage compared to FV discounts. All of the interventions were at least moderately efficacious in changing the way participants bought, cooked or ate food, with the strongest impacts observed among those who received a greater intervention dose. Finally, maintenance of newly acquired behaviours proved challenging.

It is important to understand the degree to which a program reaches those most in need of intervention [17]. Socioeconomic disadvantage is a risk factor for poor dietary quality, including low FV consumption [4, 35–39] and therefore we sought to ensure equal representation of high and low SES groups through targeted recruitment procedures. Although the sample was nearly evenly split according to neighbourhood-level SES, less than a quarter of participants were from lower income groups according to household-level measures. Disparities in study participation have also been reported in other supermarket-based studies despite the use of targeted recruitment strategies [16, 20]. SHELf had some success in reaching participants’ friends and family, with nearly one-third of participants sharing skill-building materials, 13 % sharing discounts, and an increased willingness of some participants’ partners (34 %) to consume FV due to the study. Moreover, community-wide impacts of SHELf were evident, as rollout of Coles’ fresh produce ‘super-specials’ was informed by study findings [13].

Relative to those in the control group, significantly more participants in the intervention groups reported that the study had influenced the way they purchased, cooked or consumed food at post-intervention, with no differences among intervention groups. This suggests that the skill-building intervention may have had modest positive impacts that were not captured in our earlier analysis, either because our measures were not sufficiently inclusive or because they lacked sensitivity. The former appears most likely, as those who received skill-building materials indicated that study involvement prompted them to plan shopping trips and meals more often, to incorporate more FV during cooking, to be more aware of their dietary behaviours, and to make other changes we did not previously assess. A focus on purchasing and consumption outcomes may have been responsible for similarly null findings in other educational interventions in supermarkets [15, 16], pointing to the need for broader outcome assessments to capture the potential benefits of skill-building interventions in this context. While health benefits can only be achieved through improved dietary behaviours, behaviour change is an iterative process. Thus it remains important to quantify changes in attitudes and beliefs arising from this study as they might prompt future dietary change.

In the SHELf study researchers controlled the dose of skill-building materials *delivered* to participants, however the dose *received* was under individual control. Thus, participants had to actively engage with the intervention by reading newsletters, using recipes, and completing skill-building exercises. While approximately half of participants said they read all eight newsletters and used recipes, they spent an average of just 12 min reading each newsletter, and most used between one and three of the recipes provided. Thus, a low level of engagement with skill-building materials may partly explain their lack of impact on food purchasing and consumption in the full sample, as among those who received a higher dose of intervention, positive, albeit modest, benefits were observed. At least two important implications emerge from these findings. First, newsletters and recipes may be core aspects of the skill-building intervention that should be retained in future studies, and second, a more intensive, structured and/or more engaging intervention format might be required to ensure a sufficient dose. The challenge in the former instance will be to balance intervention intensity with program reach, as participants’ time constraints may limit participation in a more intensive study. Moreover, given the low usage of the web-based forums compared to the mailed resources, we would recommend combining web- and paper-based strategies in future studies, as evidence suggests that the internet is the predominant, and an increasing source of nutrition information for consumers [40], and that messages may be reinforced when delivered through multiple channels [41].

Price reductions on FV proved extremely popular, with > 80 % of participants reporting they had accessed these, compared to < 40 % who accessed beverage discounts. Herman et al. [42] also found that FV discounts were highly accessed, with redemption rates for FV vouchers of 90 % among low-income women, whereas Waterlander et al. [16] recorded much lower redemption rates of 15–45 %. Qualitative comments further substantiated these findings, as participants overwhelmingly indicated that they appreciated and used FV discounts, whereas only a small fraction mentioned liking beverage discounts. Indeed, many deemed the beverage discounts useless because they tended not to buy carbonated beverages and water. This coincides with results from the 2011–12 Australian Health Survey, where a majority of females reported consuming fruits (65 %) and vegetables (77 %) on a given day, whereas fewer consumed regular (17 %) and diet (9 %) soft drinks [2]. These findings highlight factors underlying the absence of positive impacts of beverage discounts in our previous analysis, and may also explain similar null findings by others [43]. Comments indicating that some participants who received price discounts displaced their FV purchasing from other venues into study supermarkets to take advantage of price discounts should encourage supermarkets to offer FV at affordable prices. Such comments also suggest implications for the validity of food purchasing data in supermarket-based pricing interventions (i.e. apparent increases in FV purchasing could be partly related to a displacement effect), and will therefore be examined in a subsequent quantitative study.

Notably, although less preferred, this process evaluation revealed that the beverage discounts were not entirely without effect, as one-third of those who accessed beverage discounts reported purchasing more low-calorie carbonated beverages and water as a consequence, while those who reported accessing beverage discounts purchased an additional litre of these beverages per week. Purchase of low-calorie carbonated beverages and water did not displace purchases of high-calorie carbonated beverages among these individuals, however, as purchasing of high-calorie carbonated beverages remained stable. Thus the true impact of the beverage discounts is unclear. It is possible that these beverages are not direct alternatives and that the impacts observed were due to individuals who already regularly purchased low-calorie carbonated beverages and water using price discounts to “stock up” on these items.

The current findings support our previously reported results showing positive outcomes of FV discounts [13], as nearly two-thirds of participants indicated that they purchased more FV due to the discounts. Those who reported using the FV discounts, and who therefore received the intended FV “discount dose”, used it primarily to purchase and consume more vegetables, rather than fruit. This coincides with qualitative findings, as participants indicated a particular focus on increasing purchase and consumption of vegetables. Price reductions on FV appeared more effective than price reductions on beverages. Overall, study findings are therefore consistent with the notion that price elasticities differ across foods [44, 45]. Price discounts were clearly not sufficient to induce all participants to purchase and consume more of the discounted items, as consumers do not select foods solely based on price. Thus, multi-component interventions that simultaneously target multiple determinants of food selection and consumption are essential to improve population-level dietary behaviours.

Main outcomes from the SHELf study suggested the possibility of unintended negative consequences, as individuals in some groups increased purchase and/or consumption of high-calorie carbonated beverages at T2 and T3 [13]. We previously speculated that participants may have used saved funds to purchase more high-calorie beverages (a substitution effect), or that behaviour change activities may have unintentionally promoted increased intake of these beverages [13]. This process evaluation, however, found no evidence for these suppositions, as participants did not specifically mention using saved funds to purchase high-calorie beverages, although they did report using saved funds to purchase additional unspecified ‘foods’, which conceivably could have included high-calorie beverages. In addition, very few participants recalled any beverage-related messaging associated with the skill-building intervention, and those messages that were recalled were positive (e.g. drink more water). Thus, the observed increase in purchase and consumption of high-calorie carbonated beverages appears unrelated to the SHELf interventions.

While substituting healthier for less healthy products in the diet is desirable, simply adding more food, even healthy foods such as FV, can lead to weight gain. Studies conducted in real-world supermarket settings have reported no change in overall food expenditures [15, 16], purchase of unhealthy foods [15], or total caloric intake [43] in response to price discounts on FV and healthier beverages. At least three studies in simulated supermarket environments have, however, reported counterproductive outcomes of pricing interventions whereby discounting of healthy foods has led to higher calorie purchases [46, 47], purchase of more items overall [47] or of additional unhealthy foods [46, 48]. Qualitative comments indicating that some participants used their savings from price discounts to purchase more food overall, or more FV specifically, suggest the possibility of unintended negative consequences (i.e. increased caloric intake, although recognizing that increased FV intake confers health benefits) that should be probed in future analyses. In addition, the broader economic and health implications of food-related price reductions should be examined, as individuals could theoretically use saved funds on a variety of other more or less healthy items or activities.

While many interventions have been successful in producing short-term behaviour change, benefits often erode over time with the resumption of previous behavioural patterns [49]. Maintenance of newly established behaviours proved challenging in the SHELf study, although successes were achieved in some areas. Overall, positive perceived impacts of the interventions on the way participants bought, cooked and consumed food were largely maintained in the post-intervention period, but were no longer statistically significant, seemingly due to positive impacts of the study on these behaviours amongst participants in the control group in the post-intervention period (i.e. ~31 % of participants in all groups reported that the study had positively influenced the way they bought, cooked or ate food). Qualitative responses suggest that these positive impacts may have been partly related to the opportunity for additional self-reflection on personal eating behaviours occasioned by completion of a second survey at T2. Similarly, positive impacts of the study on the reported willingness of intervention participants’ partners to increase their FV consumption at T2 disappeared at T3 largely due to reported study-associated increases in FV consumption among partners of control group participants in the post-intervention period.

There is currently a strong focus on evaluating the impact of health promotion interventions, however less emphasis is placed on process evaluation [50]. This study was unique because of its strong design, real-world supermarket setting, grounding in an explicitly socioecological approach, multi-sectoral partnerships (i.e. Heart Foundation, Coles® supermarkets), and collection of quantitative and qualitative process-related data at multiple time points. These data can help to ascertain which aspects of the SHELf interventions were most useful, and provide more complete information for decision makers in deciding whether it is worthwhile to initiate or continue to support similar programs.

Our sample was more highly educated and had a higher income than the general population of women living in Melbourne, and therefore the generalizability of study findings is unclear. The process measures were self-reported, and may therefore be influenced by social desirability and recall biases, however these should be relatively balanced across groups. Survey questions were not validated or examined for their reliability, nor were we able to use multiple methods (e.g. individual interviews) to triangulate findings. The consistency of responses within and across questions, however, suggests that participants correctly understood the nature of the questions. Finally, we did not have purchasing and consumption data for all foods and beverages, and we were therefore limited in our ability to assess substitution effects.

## Implications and conclusions

Given the resources required to implement public health programs, the potential value of new programs should not be judged solely on the basis of their efficacy, but on a thorough and comprehensive analysis of all aspects of a program. SHELf was the first supermarket-based RCT to perform a thorough process evaluation assessing the reach, effectiveness, implementation and maintenance of a skill-building, price discount, and combined intervention. Process evaluation proved critical to a correct interpretation of study outcomes by enabling a more holistic assessment of intervention impact, and by accounting for factors such as their level of implementation. Based on these findings we have formulated several recommendations for future nutrition-related interventions in supermarkets. First, given our mixed success in recruiting disadvantaged women into the SHELf study (i.e. while 44 % of women lived in disadvantaged neighbourhoods, participants were more advantaged than average Melbournians according to individual-level SES measures) more specific actions may be required to minimize socioeconomic disparities in study participation where examining impact on disadvantaged populations is an explicit goal. Second, given that intervention impact varied according to the dose received, future studies should examine the impact of multiple doses of intervention. Third, given the importance of ensuring delivery of a sufficient intervention dose without overburdening participants, future studies could vary intervention intensity to assess feasibility and impact. Fourth, our inability to examine food substitution effects limited our conclusions related to the ultimate health impacts of our findings, and thus future studies should examine these where possible. Finally, future studies could also measure additional relevant outcomes such as change in food preparation, shopping and awareness of food selection, as these may contribute to additional behaviour change over time.

Supermarkets represent a critical, yet so far underutilized venue for influencing dietary behaviours at a population-level. This study points to the potential to leverage the power of supermarkets within food systems to virtuous ends, contributing to accomplishment of key public health objectives that might otherwise be more difficult, and take much longer to achieve. Despite modest reported effectiveness, even small changes among some population segments can shift population-level dietary intake distributions in healthier directions. It is hoped that the collective successes and failures of the SHELf intervention described herein will inform development and implementation of more effective policies and programs to optimize food selection in this setting.

## Abbreviations

FV: fruits and vegetables
RCT: randomized controlled trial
RE-AIM: reach, effectiveness, adoption, implementation, maintenance
SES: socioeconomic status
SHELf: Supermarket Healthy Eating for Life

## References

[1] Lim SS, Vos T, Flaxman AD, Danaei G, Shibuya K, Adair-Rohani H (2012). A comparative risk assessment of burden of disease and injury attributable to 67 risk factors and risk factor clusters in 21 regions, 1990–2010: a systematic analysis for the Global Burden of Disease Study 2010. Lancet.

[2] Australian Bureau of Statistics. 2014. Australian Health Survey: Nutrition First Results - Foods and Nutrients, 2011–12. http://www.abs.gov.au/ausstats/abs@.nsf/Lookup/by%20Subject/4364.0.55.007~2011-12~Main%20Features~Key%20Findings~1. Accessed January 5, 2015.

[3] Brownell KD, Kersh R, Ludwig DS, Post RC, Puhl RM, Schwartz MB (2010). Personal responsibility and obesity: a constructive approach to a controversial issue. Health Aff (Millwood).

[4] Ball K, Crawford D, Mishra G (2006). Socio-economic inequalities in women’s fruit and vegetable intakes: a multilevel study of individual, social and environmental mediators. Public Health Nutr.

[5] Shaikh AR, Yaroch AL, Nebeling L, Yeh MC, Resnicow K (2008). Psychosocial predictors of fruit and vegetable consumption in adults a review of the literature. Am J Prev Med.

[6] Thornton LE, Lamb KE, Tseng M, Crawford DA, Ball K (2015). Does food store access modify associations between intrapersonal factors and fruit and vegetable consumption? European journal of clinical nutrition.

[7] Smith KJ, McNaughton SA, Cleland VJ, Crawford D, Ball K (2013). Health, behavioral, cognitive, and social correlates of breakfast skipping among women living in socioeconomically disadvantaged neighborhoods. J Nutr.

[8] Ferranti EP, Narayan KM, Reilly CM, Foster J, McCullough M, Ziegler TR (2014). Dietary self-efficacy predicts AHEI diet quality in women with previous gestational diabetes. Diabetes Educ.

[9] Bowen DJ, Beresford SA (2002). Dietary interventions to prevent disease. Annu Rev Public Health.

[10] Beresford SA, Curry SJ, Kristal AR, Lazovich D, Feng Z, Wagner EH (1997). A dietary intervention in primary care practice: the Eating Patterns Study. Am J Public Health.

[11] McLeroy KR, Bibeau D, Steckler A, Glanz K (1988). An ecological perspective on health promotion programs. Health Educ Q.

[12] Stokols D (1992). Establishing and maintaining healthy environments. Toward a social ecology of health promotion. Am Psychol.

[13] Ball K, McNaughton S, Le H, Gold L, Ni Mhurchu C, Abbott G (2015). Influence of price discounts and skill-building strategies on purchase and consumption of healthy food and beverages: outcomes of the Supermarket Healthy Eating for Life randomized controlled trial. Am J Clin Nutr.

[14] Ball K, McNaughton SA, Mhurchu CN, Andrianopoulos N, Inglis V, McNeilly B (2011). Supermarket Healthy Eating for Life (SHELf): protocol of a randomised controlled trial promoting healthy food and beverage consumption through price reduction and skill-building strategies. BMC Public Health.

[15] Ni Mhurchu C, Blakely T, Jiang Y, Eyles HC, Rodgers A (2010). Effects of price discounts and tailored nutrition education on supermarket purchases: a randomized controlled trial. Am J Clin Nutr.

[16] Waterlander WE, de Boer MR, Schuit AJ, Seidell JC, Steenhuis IH (2013). Price discounts significantly enhance fruit and vegetable purchases when combined with nutrition education: a randomized controlled supermarket trial. Am J Clin Nutr.

[17] Glasgow RE, Vogt TM, Boles SM (1999). Evaluating the public health impact of health promotion interventions: the RE-AIM framework. Am J Public Health.

[18] Saunders RP, Evans MH, Joshi P (2005). Developing a process-evaluation plan for assessing health promotion program implementation: a how-to guide. Health Promot Pract.

[19] Escaron AL, Meinen AM, Nitzke SA, Martinez-Donate AP (2013). Supermarket and grocery store-based interventions to promote healthful food choices and eating practices: a systematic review. Prev Chronic Dis.

[20] Mhurchu CN, Blakely T, Funaki-Tahifote M, McKerchar C, Wilton J, Chua S (2009). Inclusion of indigenous and ethnic minority populations in intervention trials: challenges and strategies in a New Zealand supermarket study. J Epidemiol Community Health.

[21] Ni Mhurchu C, Blakely T, Wall J, Rodgers A, Jiang Y, Wilton J (2007). Strategies to promote healthier food purchases: a pilot supermarket intervention study. Public Health Nutr.

[22] Phipps EJ, Braitman LE, Stites SD, Singletary SB, Wallace SL, Hunt L (2015). Impact of a rewards-based incentive program on promoting fruit and vegetable purchases. Am J Public Health.

[23] Blakely T, Ni Mhurchu C, Jiang Y, Matoe L, Funaki-Tahifote M, Eyles HC (2011). Do effects of price discounts and nutrition education on food purchases vary by ethnicity, income and education? Results from a randomised, controlled trial. J Epidemiol Community Health.

[24] Australian Bureau of Statistics. 2006. Census of Population and Housing: Socio-Economic Indexes for Areas (SEIFA), Australia - Data only, 2006. http://www.abs.gov.au/AUSSTATS/abs@.nsf/DetailsPage/2033.0.55.0012006?OpenDocument. Accessed April 11, 2015.

[25] Smith LP, Ng SW, Popkin BM (2013). Trends in US home food preparation and consumption: analysis of national nutrition surveys and time use studies from 1965–1966 to 2007–2008. Nutr J.

[26] Australian Bureau of Statistics. 2009. Australian social trends. http://www.abs.gov.au/socialtrends. Accessed

[27] King DK, Glasgow RE, Leeman-Castillo B (2010). Reaiming RE-AIM: using the model to plan, implement, and evaluate the effects of environmental change approaches to enhancing population health. Am J Public Health.

[28] Jilcott S, Ammerman A, Sommers J, Glasgow RE (2007). Applying the RE-AIM framework to assess the public health impact of policy change. Ann Behav Med.

[29] Gaglio B, Shoup JA, Glasgow RE (2013). The RE-AIM framework: a systematic review of use over time. Am J Public Health.

[30] Kessler RS, Purcell EP, Glasgow RE, Klesges LM, Benkeser RM, Peek CJ (2013). What does it mean to “employ” the RE-AIM model?. Eval Health Prof.

[31] Australian Bureau of Statistics. 2011. Census of population and housing. http://www.abs.gov.au/websitedbs/censushome.nsf/home/data. Accessed January 20, 2015.

[32] Durlak JA, DuPre EP (2008). Implementation matters: a review of research on the influence of implementation on program outcomes and the factors affecting implementation. Am J Community Psychol.

[33] Patton MQ (2002). Qualitative Research & Evaluation Methods.

[34] Hawkes C (2008). Dietary implications of supermarket development: a global perspective. Develop Policy Rev.

[35] Irala-Estevez JD, Groth M, Johansson L, Oltersdorf U, Prattala R, Martinez-Gonzalez MA (2000). A systematic review of socio-economic differences in food habits in Europe: consumption of fruit and vegetables. Eur J Clin Nutr.

[36] Johansson L, Thelle DS, Solvoll K, Bjorneboe GE, Drevon CA (1999). Healthy dietary habits in relation to social determinants and lifestyle factors. Br J Nutr.

[37] Subar AF, Heimendinger J, Patterson BH, Krebs-Smith SM, Pivonka E, Kessler R (1995). Fruit and vegetable intake in the united states: the baseline survey of the five a Day for better health program. Am J Health Promot.

[38] Thornton LE, Bentley RJ, Kavanagh AM (2011). Individual and area-level socioeconomic associations with fast food purchasing. J Epidemiol Community Health.

[39] Rehm CD, Matte TD, Van Wye G, Young C, Frieden TR (2008). Demographic and behavioral factors associated with daily sugar-sweetened soda consumption in New York City adults. J Urban Health.

[40] Pollard CM, Pulker CE, Meng X, Kerr DA, Scott JA (2015). Who uses the internet as a source of nutrition and dietary information? An Australian population perspective. J Med Internet Res.

[41] Korda H, Itani Z (2013). Harnessing social media for health promotion and behavior change. Health Promot Pract.

[42] Herman DR, Harrison GG, Jenks E (2006). Choices made by low-income women provided with an economic supplement for fresh fruit and vegetable purchase. J Am Diet Assoc.

[43] Geliebter A, Ang IY, Bernales-Korins M, Hernandez D, Ochner CN, Ungredda T (2013). Supermarket discounts of low-energy density foods: effects on purchasing, food intake, and body weight. Obesity (Silver Spring).

[44] Ni Mhurchu C, Eyles H, Schilling C, Yang Q, Kaye-Blake W, Genc M (2013). Food prices and consumer demand: differences across income levels and ethnic groups. PLoS ONE.

[45] Powell LM, Chriqui JF, Khan T, Wada R, Chaloupka FJ (2013). Assessing the potential effectiveness of food and beverage taxes and subsidies for improving public health: a systematic review of prices, demand and body weight outcomes. Obes Rev.

[46] Epstein LH, Dearing KK, Roba LG, Finkelstein E (2010). The influence of taxes and subsidies on energy purchased in an experimental purchasing study. Psychol Sci.

[47] Waterlander WE, Steenhuis IH, de Boer MR, Schuit AJ, Seidell JC (2012). Introducing taxes, subsidies or both: the effects of various food pricing strategies in a web-based supermarket randomized trial. Prev Med.

[48] Giesen JC, Havermans RC, Nederkoorn C, Jansen A (2012). Impulsivity in the supermarket. Responses to calorie taxes and subsidies in healthy weight undergraduates. Appetite.

[49] Jeffery RW, Drewnowski A, Epstein LH, Stunkard AJ, Wilson GT, Wing RR (2000). Long-term maintenance of weight loss: current status. Health Psychol.

[50] Estabrooks PA, Allen KC (2013). Updating, employing, and adapting: a commentary on What does it mean to “employ” the RE-AIM model. Eval Health Prof.

